# The Sleep Connection: Exploring the Role of Composite Dietary Antioxidant Index in Sleep Quality Based on NHANES 2007-2014

**DOI:** 10.7150/ijms.114874

**Published:** 2026-01-01

**Authors:** Xiang Xu, Xin Wei, Hong Chen, Hao Gao, XingHua Chen

**Affiliations:** 1 Department of Cardiovascular Medicine, Laboratory of Chronobiology and Cardiometabolic Disease, Southwest Hospital, Army Medical University, 400038 Chongqing, China.; 2 Key Laboratory of Geriatric Cardiovascular and Cerebrovascular Disease, Ministry of Education of China, 400038 Chongqing, China.; 3 Department of Cardiovascular Medicine, Chongqing Hechuan Hongren Hospital, 401520 Chongqing, China.

**Keywords:** Composite Dietary Antioxidant Index, Sleep Duration, Sleep Trouble, Sleep Disorder, Sleep Quality

## Abstract

***Background***: Growing evidence has highlighted the critical role of oxidative stress in the pathogenesis of sleep problems. The Composite Dietary Antioxidant Index (CDAI), a holistic metric designed to assess the cumulative antioxidant capacity of dietary components, may be intricately linked to sleep quality. This study aimed to investigate the association between CDAI and sleep parameters using data from the National Health and Nutrition Examination Survey (NHANES).

***Methods***: Participants with complete data on CDAI, sleep parameters, and other essential covariates were included in this study. Weighted multivariable linear or logistic regression models were employed to examine the association between CDAI and sleep parameters (sleep duration, sleep trouble, and sleep disorder). Subgroup analyses, smooth curve fitting, threshold/saturation effect analyses, and sensitivity analyses were conducted to characterize the association's features.

***Results***: A total of 10,491 participants were finally included in the analysis. After adjusting for all potential covariates, higher CDAI levels were associated with increased sleep duration (CDAI_T3_ vs. CDAI_T1_, β=0.16, 95% CI: 0.08-0.24), while exhibiting no significant correlation with sleep trouble (CDAI_T3_ vs. CDAI_T1_, OR=1.03, 95% CI: 0.88-1.20) or sleep disorder (CDAI_T3_ vs. CDAI_T1_, OR=1.01, 95% CI: 0.79-1.28). Subgroup analyses and interaction tests indicated a more pronounced association among individuals with depression (P interaction<0.0001) and those taking sleep-modulating medications (P interaction=0.0014). Smooth curve fitting analyses identified an inverted "L"-shaped association between CDAI and sleep duration, with the inflection point of CDAI determined to be 3.2. Within this threshold, each 1-unit increase in CDAI was associated with a 0.04-hour (95% CI: 0.02-0.05) increase in sleep duration, whereas no association was observed beyond this point. Finally, sensitivity analyses, which excluded individuals with extreme energy intakes or depression, confirmed a consistent and robust association between CDAI and sleep duration.

***Conclusion***: The association between CDAI and sleep duration exhibits a positive, inverted “L”-shaped pattern with a saturation effect. Further prospective or longitudinal cohort studies with larger sample sizes and longer follow-up periods are needed to evaluate its practical value for clinical interventions.

## Introduction

Sleep is an innate and reversible state primarily regulated by neurobiological mechanisms, representing an essential physiological aspect of human existence crucial for maintaining optimal health 1. The major characteristic of this state is the reduction of perception to external stimuli and cessation of motor activity, which distinguish it from wakefulness. Sleep quality can be modulated by various factors, including physical activity, dietary patterns, genetic predispositions, and environmental influences 2,4. In fact, these determinants interact in complex ways to shape the bidirectional association between sleep and health outcomes. On the one hand, adequate sleep confers profound benefits: it reduces energy consumption, restores brain homeostasis, modulates the immune response, and consolidates memory 1. On the other hand, however, sleep disorders are intimately linked to the pathogenesis of multiple diseases—ranging from depression 5 and arterial stiffness 6 to cardiovascular disease 7, infectious diseases 8, and even cancer 9. Evidence from previous studies is striking: up to 50% of children 10, 27.1% of adults 11, and 50% of older Americans 12 experience varying degrees of sleep disturbances. This highlights sleep disorders as a critical public health issue, imposing a substantial burden on both individuals and healthcare systems. In the contemporary era, dramatic shifts in work schedules, lifestyle patterns, and dietary habits are likely exacerbating sleep problems across all age groups. Therefore, unraveling the key factors influencing sleep quality is not only scientifically imperative but also essential for developing targeted interventions to mitigate this growing health crisis.

In recent years, increasing evidence has revealed the close association between oxidative stress and sleep disorders 13-15. Oxidative stress arises from the imbalanced status between production and scavenging of reactive oxygen species (ROS) 16. Numerous dietary components with antioxidant properties have been reported to benefit humans and experimental animals through various mechanisms. A recent study using flies and mice demonstrated that sleep deprivation leads to severe ROS accumulation and oxidative stress in the gut, causing death in both species. Interestingly, oral administration of antioxidant compounds or overexpression of antioxidant enzymes in the gut enabled flies to maintain a normal lifespan even under conditions of minimal or no sleep 17. In humans, a prospective, double-blinded randomized controlled trial showed that vitamin E supplementation can serve as an effective alternative treatment for alleviating chronic insomnia disorder 18. Further evidence from other studies suggests that individuals with short sleep duration who consume high levels of dietary antioxidant vitamins have a significantly lower risk of obesity than those with normal sleep duration 19. Additionally, clinical studies have shown that antioxidant dietary supplements (e.g., vitamin C, luteolin, magnesium, B6, B12, folic acid, etc.) can significantly improve sleep problems and related complications arising from various factors, such as nighttime traffic noise, sleep deprivation, and obstructive sleep apnea 20-22. Collectively, these findings highlight the pivotal role of antioxidants in enhancing sleep quality. Therefore, a quantifiable evaluation index of antioxidant components is needed to facilitate improvements in sleep quality.

The Composite Dietary Antioxidant Index (CDAI) is a comprehensive and quantifiable indicator that integrates information on multiple dietary antioxidants (including vitamins A, C, E, zinc, selenium, and manganese) to assess an individual's overall dietary antioxidant intake profile. Numerous studies have established close associations between CDAI and various health outcomes, including hypertension 23, all-cause and cardiovascular mortality 24, sarcopenia 25, cancer risk 26, osteoporosis 27, among others. Given the intimate link between oxidative stress and sleep, a potential association may exist between CDAI and sleep quality. However, whether such a relationship actually exists remains unelucidated. Therefore, this study aimed to explore the association between CDAI and sleep quality using data from the NHANES database, with the goal of providing dietary guidance to improve sleep quality.

## Materials and Methods

### Study population

The National Health and Nutrition Examination Survey (NHANES) is a comprehensive research program conducted by the Center for Disease Control and Prevention (CDC), which aims to evaluate the health and nutritional well-being of both adults and children in the United States 28. Started in the early 1960s, the NHANES program has conducted a series of surveys concerning different population groups, health problems, and nutrition statuses to meet the emerging public needs. The survey within NHANES program was conducted annually and a sample of about 5,000 persons each year was examined, and the demographic, socioeconomic, dietary, and health-related information was collected. The approval for protocols was already granted by the ethical review board of National Center for Health Statistics (NCHS). All researchers who have meet the requirements can utilize the database for study purposes with no need to submit additional applications 29. The information sensitive information of all participants was anonymized and individually unrecognizable 29. The informed consents were signed by all participants.

**Figure 1:**
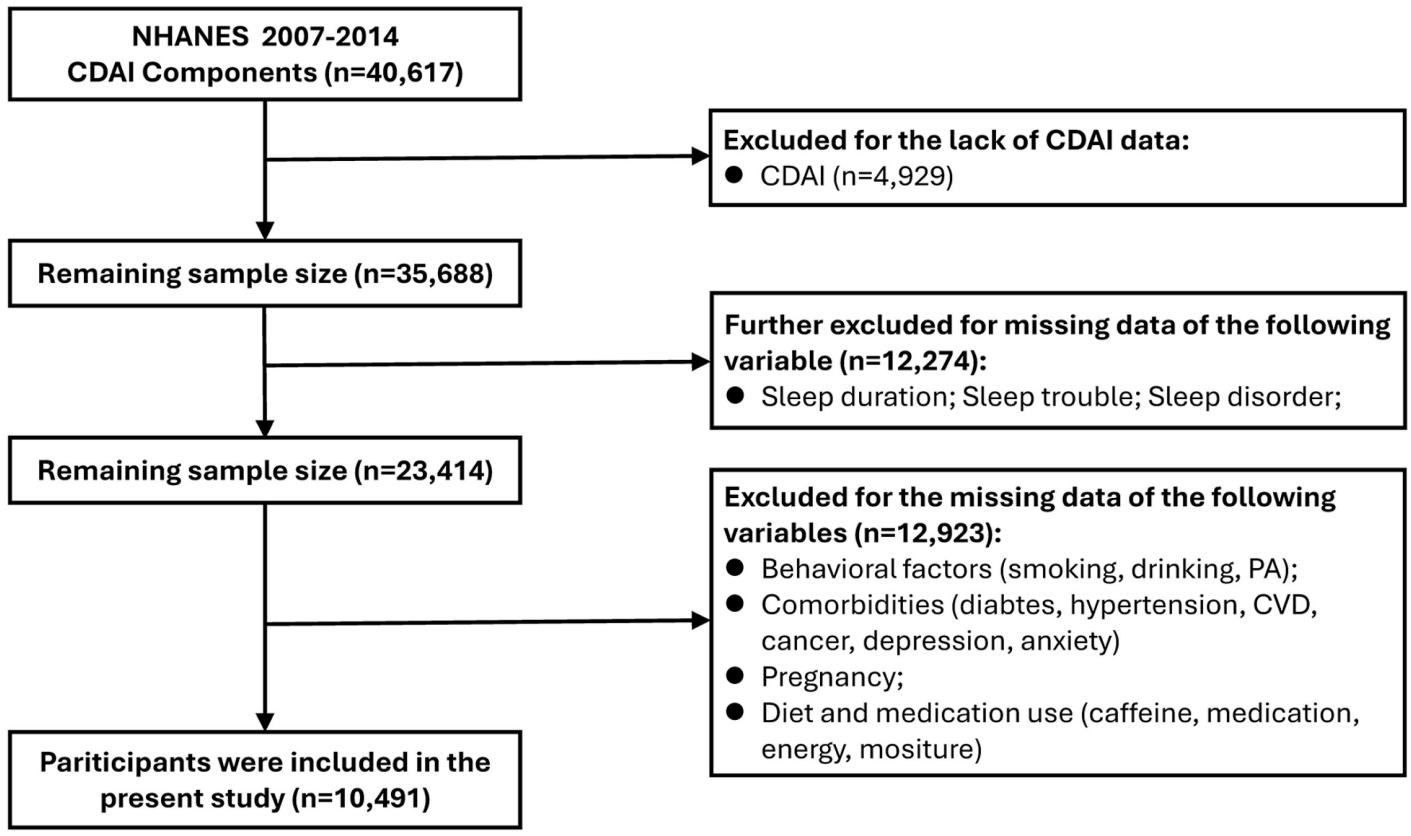
** Flowchart of participants screening. *Abbreviations:*** CDAI, composite dietary antioxidant index; PIR, poverty income ratio; BMI, body mass index.

The sample selection flowchart is presented in **Figure 1**. In brief, 40,617 participants from four NHANES cycles (2007-2008, 2009-2010, 2011-2012, and 2013-2014) with available dietary CDAI component data were initially enrolled. Subsequently, 4,929 participants with missing CDAI data were excluded, followed by 12,274 participants lacking sleep parameter data. Further exclusions included participants with incomplete information on: 1) behavioral factors (smoking, alcohol consumption, physical activity); 2) comorbidities (diabetes, hypertension, cardiovascular disease, cancer, depression, anxiety); 3) pregnancy status; and 4) dietary/medication use (caffeine intake, medication use, energy, moisture). Finally, 10,491 participants were included in the analysis.

### CDAI calculation and exposure definition

As previously reported 30-32, CDAI was calculated using a validated method developed by Wright with minor modifications. The core logic of the CDAI calculation is to simplify the highly correlated group of antioxidant nutrients via principal component analysis (PCA), then integrate the independent antioxidant nutrients, and finally form a comprehensive index to quantify the overall dietary intake level of antioxidant substances. This method not only retains information about the synergistic effects of different types of antioxidant nutrients but also avoids the limitations of single-nutrient analysis, making it more suitable for evaluating the association between complex dietary patterns and health outcomes (e.g., lung cancer risk 32).

This method assesses six food-derived minerals and vitamins associated with antioxidant processes: selenium, zinc, vitamins A, C, E, and carotenoids. CDAI is computed via the following formula:



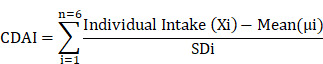



Here, Xi denotes the daily intake of antioxidant i, and μi is the mean intake of Xi across the entire cohort, and SDi represents the standard deviation for μi.

### Sleep quality measures and outcome definitions

As previously described 33,34, three dimensional questions were utilized to evaluate participants' sleep quality. The information for these questions was collected, in the home, by professionally trained interviewers by utilizing the computer-assisted personal interviewing (CAPI) system, which is equipped with built-in consistency checks to minimize data entry errors and ensure data accuracy. Besides, online help screens are also available to assist interviewers in understanding and defining key terms mentioned in the questionnaire.

Specifically, the question of “(SLD010H) How much sleep do you get (hours)?” was used to get the participants' sleep duration. Besides, those participants who responded “yes” to the question of “(SLQ050) Ever told doctor had trouble sleeping?” were considered to have sleep trouble, while participants who answered “yes” to the question of “ (SLQ060) Ever told by doctor have sleep disorder?” were considered to have sleep disorder.

### Covariates

Based on previous studies on sleep, essential covariates were considered, including age (<40, 40-60, >60), gender (male, female), race (Mexican American, Other Hispanic, Non-Hispanic White, Non-Hispanic Black, Other Race), education (less than high school, high school or equivalent, college or above), marital status (married, never married, others), PIR (<1.3, 1.3-3.5, >3.5), BMI (<25, 25-30, >30), smoking status (never, former, current), alcohol consumption (mild, moderate, heavy), physical activity (PA; low, high), diabetes (no, yes), hypertension (no, yes), CVD (no, yes), cancer (no, yes), depression (no, yes), anxiety (no, yes), pregnancy (no, yes), medication use (no, yes), as well as caffeine intake, energy intake, and moisture. Age was categorized into three groups as previously described 35: <40, 40-60, and >60. Race included five categories: Mexican American, other Hispanic, Non-Hispanic White, Non-Hispanic Black, and other race. Education was divided into three subgroups 35: less than high school, high school or equivalent, and college or above. Similarly, marital status included three categories 35: married, never married, and others. PIR was classified into three groups following previous studies 35: <1.3, 1.3-3.5, and >3.5. Moreover, BMI was divided into three groups according to the World Health Organization (WHO) criteria 36: normal/low body weight (<25 kg/m²), overweight (25-30 kg/m²), and obese (>30 kg/m²). Smoking status was classified into three subgroups based on self-reported questionnaire responses: never, former, and current smokers 37. Specifically, individuals who smoked fewer than 100 cigarettes in their lifetime were defined as never smokers, those who had smoked at least 100 cigarettes but had quit smoking were classified as former smokers, and those who currently smoke and have smoked over 100 cigarettes were identified as current smokers. Alcohol consumption status was categorized into three levels based on average daily alcohol intake in the past year: mild drinking (females ≤1 drink/day; males ≤2 drinks/day), moderate drinking (females 1-3 drinks/day; males 2-4 drinks/day), and heavy drinking (females ≥4 drinks/day; males ≥5 drinks/day) 38. Physical activity (PA) information was assessed using the Global Physical Activity Questionnaire (GPAQ), and weekly total metabolic equivalent (MET) values were used to quantify PA 39-41. According to previous studies 42,43, a cut-off value of 500 MET-minutes/week was applied to define PA levels: low PA (<500 MET-minutes/week) and high PA (≥500 MET-minutes/week). Diabetes can be defined if participants meet any one of the following criteria 44: 1) self-reported diabetic diagnosis by physicians, or use of medication/insulin for diabetes treatment; 2) hemoglobin A1C (HbA1C) ≥6.5% or fasting plasma glucose (FPG) ≥126 mg/dL (≥7.00 mmol/L); and 3) two-hour oral glucose tolerance test (OGTT) ≥11.10 mmol/L, random blood glucose ≥11.10 mmol/L. The diagnosis of hypertension can be established by meeting any one of the following criteria 45: 1) self-reported hypertension diagnosis by clinicians, or the use of antihypertensive medication currently for treatment; 2) with average systolic blood pressure (SBP) more than 130 mmHg, or average diastolic blood pressure (DBP) over 80 mmHg. The diagnosis of cardiovascular disease (CVD) 46 was established if individuals responded "yes" to any of the following five clinical questions: "Have you ever been told by a physician that you had coronary heart disease (CHD), congestive heart failure (CHF), angina, a heart attack, or a stroke?". For cancer diagnosis, individuals were classified as having cancer if they responded "yes" to the questionnaire item "MCQ220 - Ever told by a doctor that you had cancer or malignancy.". A nine-item depression screening instrument [namely the Patient Health Questionnaire (PHQ-9)] was used to assess depressive symptoms. As previously described, a PHQ-9 score ≥10 was used to define clinically significant depression 47. The anxiety state was assessed by the question asking "During the past 30 days, for about how many days have you felt worried, tense, or anxious?", and individuals were further categorized as without anxiety (felt anxious for 0-6 days per month) and with anxiety (felt anxious for 7-30 days per month) 48. Pregnancy status was assessed by the question "RHD143 - Are you pregnant now?", and those who answered "yes" were defined as pregnant. Dietary intake data (e.g., energy, caffeine, moisture) were determined by two 24-hour dietary recall interviews: the first was collected in person at the Mobile Examination Center (MEC), and the second was conducted by telephone 3-10 days later. The status of medication use was determined by the following two questions: "RXDUSE - Taken prescription medicine in the past month" and "RXDDRUG - Generic drug name". Individuals were categorized into two groups (yes/no) based on whether medications affected sleep quality: (1) those taking medications that could impact sleep quality, and (2) those not taking any medications or taking medications without sleep-related effects.

### Statistical analysis

Sample weight calculation, clustering, and stratification were taken into account for all analyses conducted in this study. The data analysis process involved utilizing the complex sampling weight calculation provided by the NHANES analysis guide. The baseline characteristics were presented based on the tertiles of CDAI, and continuous variables were presented as median and interquartile ranges (IQR) for their skewed normal distribution characteristics while categorical variables were showed as frequency (percentage). The differences in baseline characteristics across CDAI tertile groups were evaluated using the Rao-Scott chi-squared test or Kruskal-Wallis test for categorical and continuous variables, respectively.

Weighted multivariable regression models were used to explore the association between CDAI and sleep parameters, including logistic regression for categorical outcomes (sleep trouble and sleep disorder) and linear regression for the continuous outcome (sleep duration). Three models were established with increasing covariate adjustment: model 1 was unadjusted (no covariates), model 2 was adjusted for age, gender, race, education, marital status, and PIR, and model 3 further adjusted for BMI, smoking status, alcohol consumption, physical activity (PA), diabetes, hypertension, CVD, cancer, depression, anxiety, caffeine intake, medication use, energy intake, and moisture intake on the basis of model 2. Moreover, adjusted restricted cubic spline (RCS) analyses were used to explore the potential non-linear relationship between CDAI and sleep parameters, and the “segmented” package was further used to determine the inflection point once a non-linear relationship was identified as previously described 49. Additionally, subgroup analyses and interaction tests were performed to examine the robustness and consistency of the association between CDAI and sleep quality across subgroups. When significant interaction effects were identified in the tests, subsequent stratified smooth curve fitting analyses were conducted to explore the associations between CDAI and sleep parameters in each subgroup. Finally, sensitivity analyses were conducted to further validate the association between CDAI and sleep parameters by excluding participants with extreme energy intake and those who were pregnant. R (version 3.5.3) were used to conduct statistical analyses. P-values<0.05 were considered statistically significant.

## Results

### Baseline characteristics of the study population

A total of 10,491 participants were finally included in this study, with baseline characteristics based on CDAI tertiles presented in **Table 1**. As shown in **Table 1**, compared with the lowest CDAI tertile group, individuals in the highest CDAI tertile group had significantly higher PIR, BMI, and intakes of caffeine, moisture, and energy. They were more frequently male, Non-Hispanic White, educated at college or above, married, never-smokers, mild drinkers, and physically active. Additionally, they were less likely to have diabetes, hypertension, CVD, depression, or anxiety, and less likely to use medications affecting sleep quality (all P < 0.05).

### Association between CDAI and sleep parameters

**Figure 2:**
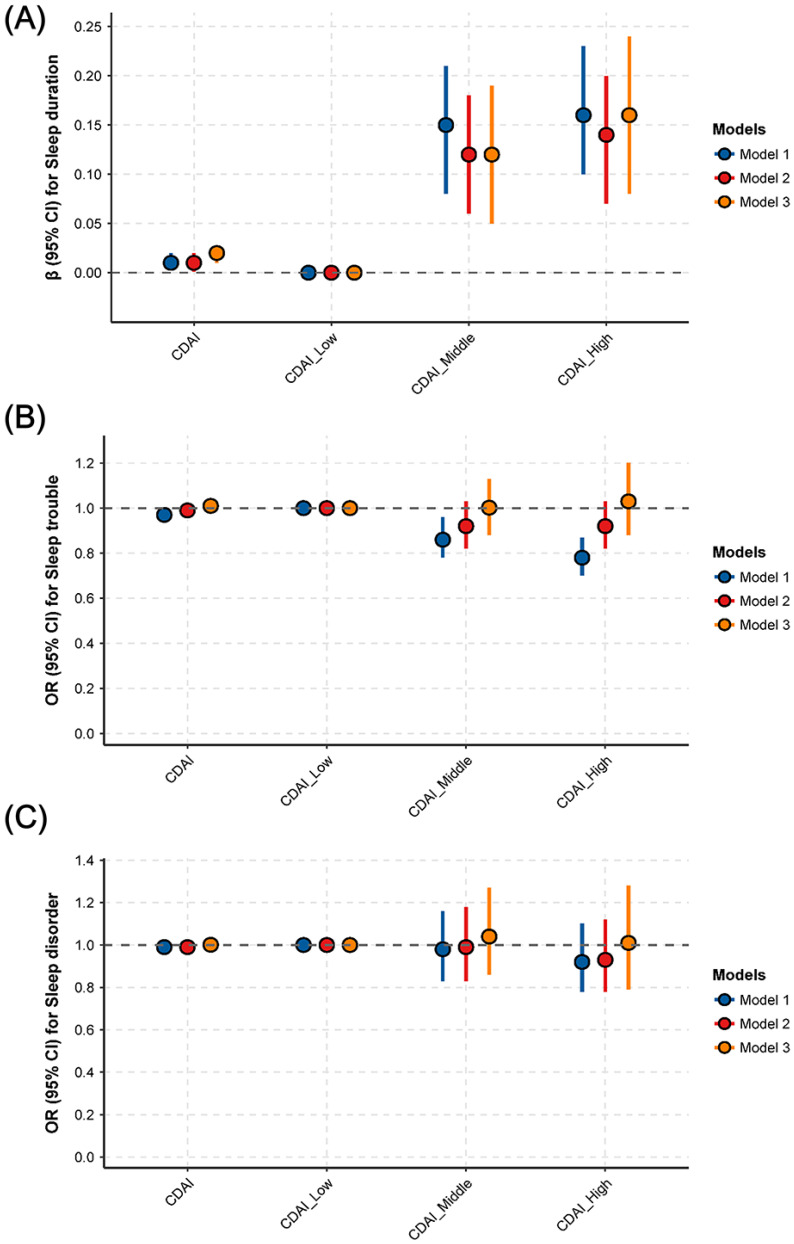
** Associations between CDAI and sleep duration, sleep trouble and sleep disorder. (A)** sleep duration; **(B)** sleep trouble; **(C)** sleep disorder. ***Abbreviations:*** OR, odds ratio; CI, confidence intervals; CDAI, composite dietary antioxidant index; PA, physical activity; CVD, cardiovascular disease; BMI, body mass index; PIR, ratio of family income to poverty. ***Note:*** Model 1: adjusted for no covariates. Model 2: adjusted for variables of Age, Gender, Race, Education, Marital status, PIR. Model 3: adjusted for variables of age, gender, race, education, marital status, PIR, BMI, smoking, drinking, PA, diabetes, hypertension, CVD, cancer, depression, anxiety, caffeine, medication use, energy, and moisture.

The association between CDAI and sleep parameters is presented in **Fig. 2**. After adjusting for all potential covariates, a positive and significant association was identified between CDAI and sleep duration: a one-unit increase in CDAI (as a continuous variable) was associated with a 0.02-hour increase in sleep duration (95% CI: 0.01-0.02, P = 0.0011) (**Fig. 2A, STable 1**). Similarly, compared with the lowest CDAI tertile group, the highest CDAI tertile group had a 0.16-hour longer sleep duration (95% CI: 0.08-0.24, P = 0.0001) (**Fig. 2A, STable 1**). However, in the fully-adjusted model (model 3), no significant associations were found between CDAI and sleep disturbance (**Fig. 2B, STable 1**) or sleep disorder (**Fig. 2C, STable 1**) (all P > 0.05).

**Figure 3:**
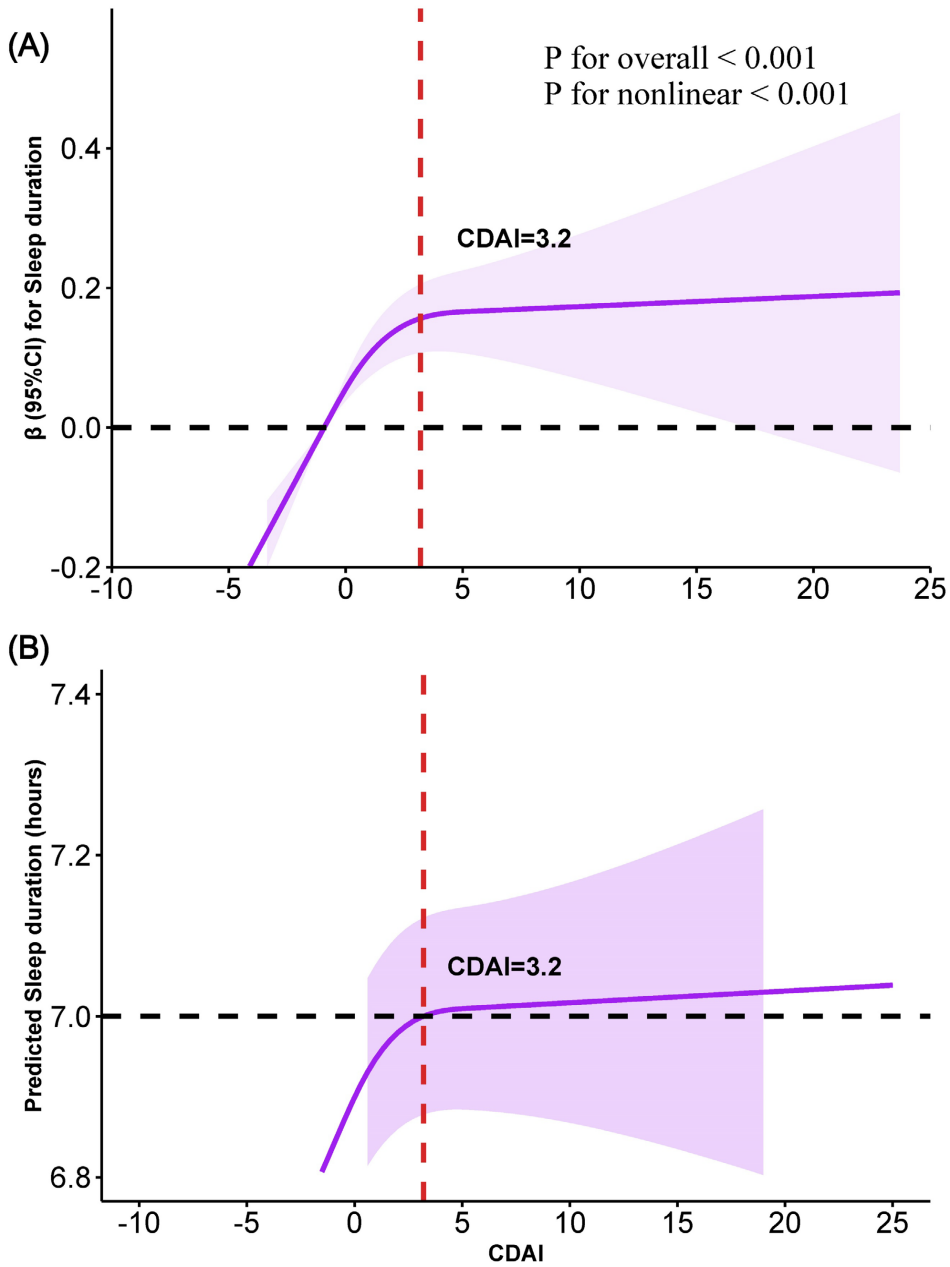
** Association between CDAI and sleep duration. (A)** Identifying the nonlinear association between CDAI and sleep duration using RCS analysis. **(B)** Smooth curve fitting showing the relationship between CDAI and predicted sleep duration (in hours). Variables of age, gender, race, education, marital status, PIR, BMI, smoking, drinking, PA, diabetes, hypertension, CVD, cancer, depression, anxiety, caffeine, medication use, energy, and moisture were adjusted during analyses.

Moreover, adjusted restricted cubic spline (RCS) analysis was used to explore the potential non-linear association between CDAI and sleep duration. As shown in **Fig. 3**, a significant positive non-linear association (inverse “L” shape) was found between CDAI and sleep duration (P for overall < 0.001, *P* for nonlinearity < 0.001). Using the "segmented" package, an inflection point of 3.2 for CDAI was identified (**Fig. 3**). Below this threshold, a significant positive relationship existed: each 1-unit increase in CDAI was associated with a 0.04-hour increase in sleep duration (95% CI: 0.02-0.05, P < 0.0001) (**Table 2**). However, no significant association was observed between CDAI and sleep duration after exceeding this point (95% CI: -0.01 to 0.01, P=0.8807) (**Table 2**).

### Subgroup analyses

**Figure 4:**
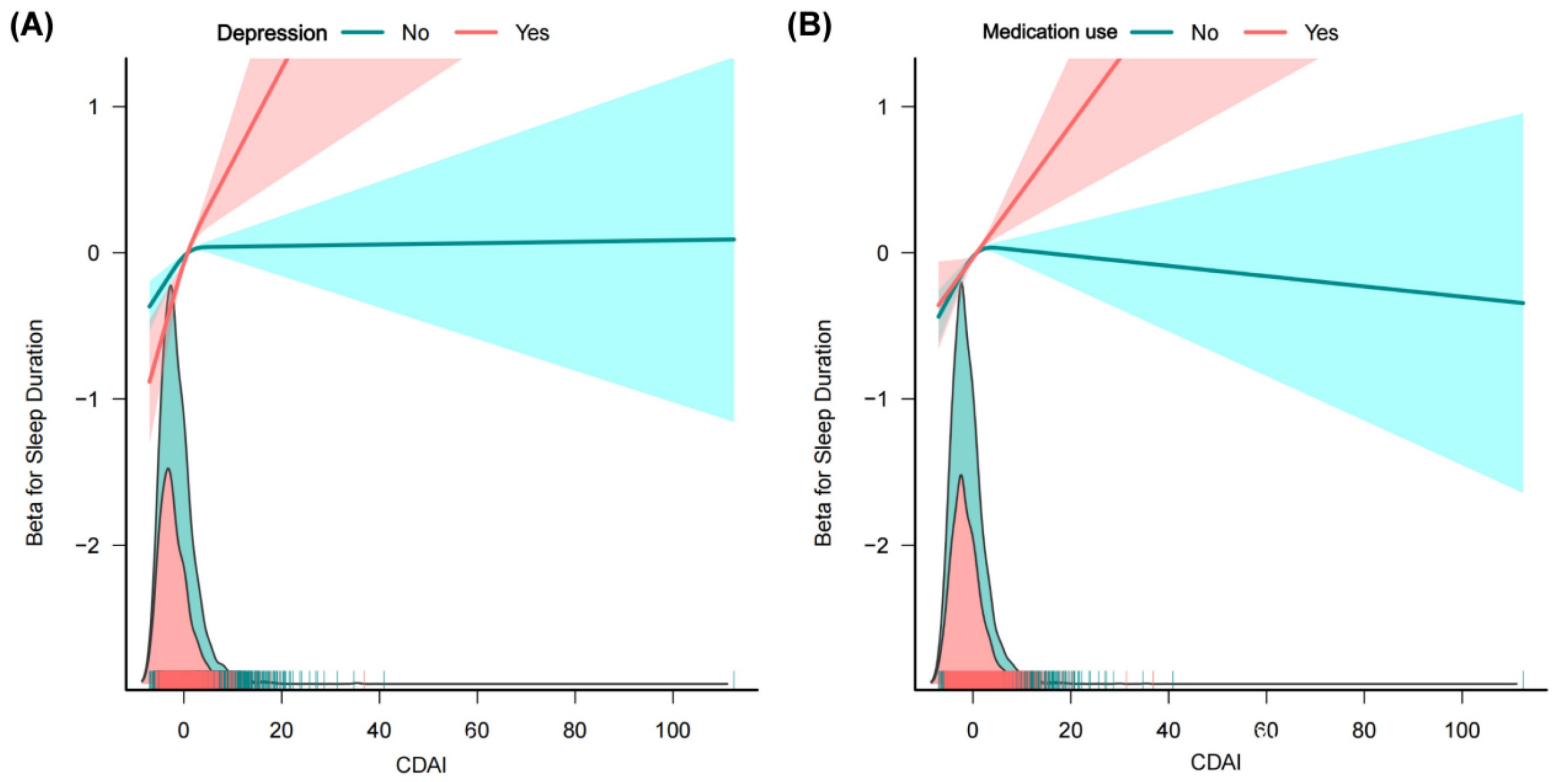
** Associations between CDAI and sleep duration in different subgroups. (A)** Depression; **(B)** Medication use.

To further explore the robustness of the association between CDAI and sleep duration, subgroup analyses and interaction tests were conducted. As shown in **Table 3**, a more pronounced association was observed in individuals aged <40 years, with higher education, never married, PIR <1.3, BMI >30 kg/m^2^, never smokers, heavy drinkers, low physical activity (PA) levels, without diabetes/CVD/hypertension/cancer/anxiety, and those with depression or taking medications affecting sleep quality (**Table 3**). Moreover, the CDAI-sleep duration association was modified by depression (P for interaction < 0.0001) and medication use (P for interaction = 0.0014), with stronger associations observed in individuals with depression or using sleep-affecting medications (**Table 3**). These findings were further corroborated by stratified restricted cubic spline (RCS) analyses, as illustrated in **Fig. 4**.

### Sensitivity analyses

**Figure 5:**
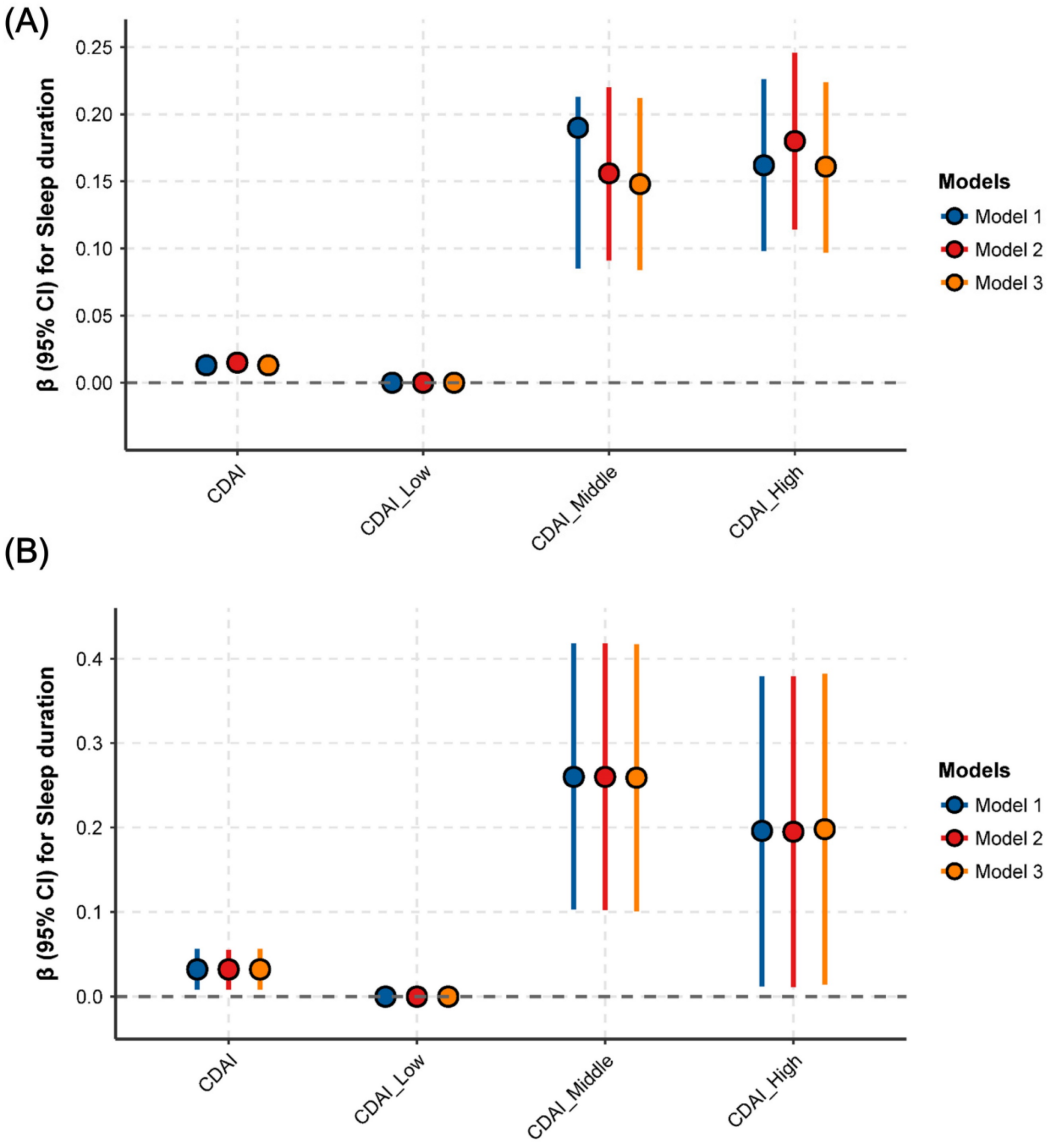
** Sensitivity analyses. (A)** Association between CDAI an sleep duration after excluding participants with energy extremes; **(B)** Association between CDAI an sleep duration after excluding participants with pregnancy. ***Note:*** variables adjusted in sensitivity analyses were consistent with Model 3 in Figure 2.

Given the evidence from prior studies on the effects of energy intake and pregnancy on sleep, sensitivity analyses were performed to validate the association between CDAI and sleep duration across subgroups 50-52. In most epidemiologic studies, participants with implausible energy intakes (i.e., extreme values) are excluded, typically defined as <500 kcal/day or >3,500 kcal/day 53. Based on this criterion, 335 participants were identified with extreme energy intakes (SFig. 1). Subsequent multivariable linear regression models were used to assess the association's robustness after excluding these outliers. The association between CDAI and sleep duration remained consistent among participants with plausible energy intakes (**STable 2, Fig. 5A**). Similarly, consistent results were observed after excluding participants with pregnancy (**STable 3, Fig. 5B**). Collectively, these findings—along with prior results—confirm a stable association between CDAI and sleep duration across diverse subgroups.

## Discussion

Our study identified a significant positive nonlinear association between CDAI and sleep duration after adjusting for all potential covariates. This association was particularly pronounced in individuals with depression or taking sleep-affecting medications. Using threshold and saturation effect analysis, we identified an inflection point of 3.2 for CDAI. Below this threshold, each 1-unit increase in CDAI was associated with a 0.04-hour increase in sleep duration; however, no further improvement in sleep duration was observed above this inflection point. Collectively, our results suggest that higher CDAI levels may beneficially impact sleep duration, particularly below the identified threshold.

Although the specific mechanisms by which CDAI components improve sleep quality remain unclear, their antioxidant, redox modulation, and anti-inflammatory properties may play a crucial role. Previous studies have indicated that Nrf2 was a pivotal hub linking sleep to oxidative stress, inflammation, and circadian rhythms 54,55. Insufficient sleep or sleep-disordered breathing are reported to suppress Nrf2 activity, thereby reducing antioxidant defenses and elevating inflammation—effects that further deteriorate sleep quality and perpetuate a vicious cycle 54. In contrast, activation of Nrf2 via pharmaceutical, nutritional, or genetic modulation can partially reverse these adverse outcomes, with animal studies showing improved sleep architecture and cognitive function 54. A growing body of evidence suggests that most key nutrients comprising the CDAI exert their neuroprotective effects primarily through activation of the Nrf2 signaling pathway. Vitamin C has been shown to upregulate Nrf2 and its downstream targets, such as HO-1 and NQO1, thereby attenuating oxidative stress 56,57. Similarly, δ- and γ-tocopherol isoforms of vitamin E can directly promote the nuclear translocation of Nrf2 and enhance the expression of antioxidant enzymes 58. Selenium contributes to Nrf2 activation by inducing target genes such as GPX4, which plays a critical role in protecting neural tissues against ferroptosis 59. Zinc not only facilitates Nrf2 nuclear translocation but also suppresses ROS accumulation, consequently inhibiting activation of the NLRP3 inflammasome 60-62. Carotenoids—including β-carotene, α-carotene, lutein, and lycopene—are also capable of inducing Nrf2 expression and boosting antioxidant enzyme levels; certain carotenoids, such as astaxanthin, may even reinforce Nrf2 activity through epigenetic regulation 63,64. Taken together, these findings indicate that diets enriched in vitamin C, δ/γ-tocopherols, selenium, zinc, and carotenoids (namely diets with higher CDAI level) can effectively enhance antioxidant defenses and mitigate neuroinflammation via Nrf2-dependent mechanisms. This mechanistic framework may partly explain the observed association between higher CDAI levels and improvements in sleep duration and quality.

In this study, a stronger association between CDAI and sleep duration was observed in individuals with depression, indicating that participants in this subgroup were more likely to derive greater sleep benefits at higher CDAI levels. Depression is widely recognized not merely as a psychological disorder but as a physiological disease involving multisystem dysregulation, characterized principally by oxidative stress imbalance and chronic low-grade inflammation. These pathological alterations critically disrupt sleep regulatory pathways. Specifically, depressed individuals exhibit hyperactivation of the hypothalamic-pituitary-adrenal (HPA) axis, which disrupts circadian melatonin secretion and thereby disturbs sleep-wake rhythms 65,66. Additionally, elevated serum levels of proinflammatory cytokines (e.g., CRP, IL-6, TNF-α) in depression cross the blood-brain barrier to directly inhibit sleep-regulatory functions in the prefrontal cortex and hypothalamus, leading to sleep fragmentation and reduced slow-wave sleep 67-69. Concurrently, impaired antioxidant defenses (e.g., decreased SOD and GPx enzymatic activity) in depression enhance reactive oxygen species (ROS), inducing neuronal lipid peroxidation and DNA damage that compromise the functional integrity of sleep-wake regulatory nuclei 70-72. Collectively, these pathological mechanisms create a "nutritional intervention window" for depressed patients: dietary intake of antioxidant-rich compounds (e.g., vitamins A, C, E) can more effectively neutralize free radicals and mitigate inflammation, thereby reconstructing a sleep-conducive molecular environment. This may explain the stronger association between CDAI and sleep duration observed in individuals with depression.

Similar enhanced associations between CDAI and sleep duration were observed among individuals using sleep-modulating medications, indicating that higher antioxidant nutrient intake may confer greater sleep benefits to this subgroup. Most sleep-modulating medications rely on multiple mechanisms to regulate neurotransmitter milieu—such as enhancing neuronal GABA receptor sensitivity or mimicking endogenous melatonin—processes that can be modulated by antioxidant nutrient levels. For example, benzodiazepines and non-benzodiazepine hypnotics (e.g., zolpidem, zopiclone) primarily reduce neuronal excitability by potentiating GABAergic neurotransmission, thereby lengthening sleep duration 73. Notably, low oxidative stress levels enhance neuronal GABA receptor sensitivity to these drugs, further prolonging sleep. Additionally, melatonin receptor agonists like ramelteon improve sleep quality by mimicking endogenous melatonin 74,75. When CDAI elevation alleviates oxidative damage, pinealocyte function recovers partially, increasing endogenous melatonin secretion and creating a synergistic effect with exogenous agonists. These mechanistic interactions between sleep-modulating drugs and antioxidant nutrients may underlie the stronger CDAI-sleep duration association in this population.

The study further revealed that the relationship between the Composite Dietary Antioxidant Index (CDAI) and sleep duration exhibits a clear saturation effect, with the inflection point occurring at a CDAI of 3.2. When CDAI is below 3.2, each additional unit of CDAI is associated with an increase of 2-8 minutes of sleep, gradually moving sleep duration toward the 7-hour mark. Once CDAI reaches 3.2, sleep duration stabilizes around 7 hours, indicating that the effect has plateaued. This finding is important because a 7-hour sleep duration has been repeatedly confirmed by epidemiological research as a “healthy threshold” that is strongly linked to lower risks of all-cause mortality, cardiovascular disease, biological aging, metabolic syndrome, and other adverse health outcomes 76-78. For individuals with a CDAI below 3.2—i.e., whose sleep duration has not yet reached the 7-hour threshold—each unit increase in CDAI, although translating only into minute-level gains in sleep, is not trivial. Instead, it represents a critical incremental step that helps them cross the “7-hour healthy threshold.” In a context where sleep deprivation is common, this “small effect driving threshold breakthrough” holds considerable public-health relevance. Moreover, while CDAI is formally a composite index of dietary antioxidant intake, it essentially serves as a measurable proxy for a “multidimensional healthy lifestyle.” Its association with sleep duration reflects the combined influence of “optimized diet + regular sleep schedule + moderate physical activity + low-risk behaviors.” From this perspective, the sleep benefit linked to each unit increase in CDAI does not arise from a single dietary factor; rather, it signals the synergistic improvement of overall health-behavior patterns. When an individual's CDAI rises and sleep duration approaches the 7-hour threshold, this change indicates broader lifestyle optimization. Consequently, the chain of “behavior cluster - sleep threshold - health outcomes” carries far greater clinical significance than the modest minute-level sleep gain alone. It suggests that enhancing diet (raising CDAI) can catalyze comprehensive lifestyle improvements, indirectly steering population sleep duration toward the healthy threshold and thereby reducing the risk of multi-system diseases. This linkage has substantial public-health implications, surpassing the impact of isolated “minute-level sleep effects.” Briefly, although the effect size appears modest in terms of minutes, when considered alongside the saturated 7-hour healthy threshold and the lifestyle-proxy nature of CDAI, the findings retain clear clinical relevance and population-health significance. They can aid in identifying high-risk groups with insufficient sleep and in guiding holistic lifestyle interventions that begin with dietary modifications.

The sensitivity analyses in this study yielded consistent results. By excluding participants with implausible energy intake (a well-documented source of dietary reporting bias) and those with depression, the CDAI-sleep duration association persisted. This indicates that the association is not a statistical artifact caused by extreme caloric reporting errors but reflects the true biological effects of dietary antioxidants. These results, together with the identification of a nonlinear threshold and subgroup consistencies, collectively validate the fundamental role of CDAI in sleep health. Additionally, the findings reveal that optimizing CDAI through dietary strategies could complement existing sleep interventions.

Our study found a significant negative correlation between CDAI and sleep duration, while no significant association was observed between CDAI and sleep trouble or sleep disorder. This inconsistency may stem from the following aspects. First, sleep duration is a relatively objective and easily quantifiable indicator, whereas the assessment of sleep trouble/sleep disorder is more subjective and more susceptible to the influence of multiple strong confounding factors (e.g., transient emotional states, comorbid pain, and psychosocial stress). These factors may mask the relatively weak effect of dietary inflammatory potential. Second, although a comprehensive set of covariates were adjusted, residual factors such as chronic social stress were not included. These factors have been reported to be closely associated with subjective sleep quality and may have obscured CDAI's effect 79,80. Third, CDAI is hypothesized to exert its effects through modulating inflammatory activity 81,82, and such inflammatory activity primarily impacts sleep structure by reducing deep sleep duration—with a more indirect influence on subjective sleep quality. This mechanistic pathway specifically aligns with our observation that CDAI associates only with sleep duration. The association between CDAI and sleep duration thus suggests that inflammatory activity may affect participants' total sleep duration by influencing sleep structure, but its impact on sleep quality still requires further research to verify. Future longitudinal studies should incorporate more comprehensive psychological assessments and objective sleep monitoring (e.g., actigraphy, polysomnography) to clarify the interactions among inflammation, psychological factors, and different sleep dimensions.

Our study offers several key advantages. First, it was based on a nationally representative NHANES population, which provided an adequate sample size and reliable dataset to uncover associations between CDAI and sleep quality. Second, sleep quality among enrolled participants was systematically assessed through subjective and objective measures, including self-reported sleep trouble, sleep disorders, and sleep duration. Third, adjustment for well-known potential confounding variables in sleep research enabled us to derive more accurate results. Additionally, subgroup analyses were conducted to validate the CDAI-sleep quality correlation across different populations, while interaction analyses explored how confounding variables influenced the outcomes. Finally, our findings revealed that individuals with a CDAI level ≥3.2 were more likely to have a predicted sleep duration of at least 7 hours—an insight with significant implications for informing daily lifestyle choices and clinical practices.

Several limitations of this study should be acknowledged. First, some variables were based on self-reported data (e.g., sleep parameters, alcohol consumption, physical activity, etc.), which may introduce recall and social desirability biases into the study. Especially, it is worth noting that the assessment of sleep quality in the NHANES database is not derived from objectively measured instrument data but relies on a few limited and subjective questions regarding sleep habits and sleep disorders. Consequently, this simplified evaluation method cannot fully capture key dimensions of sleep quality, such as the severity of insomnia, sleep latency (time taken to fall asleep), or sleep efficiency. The use of such subjectively evaluated key outcome variables may introduce a certain degree of bias into our findings. Therefore, future studies should employ objective sleep monitoring devices (e.g., actigraphy, polysomnography) to assess sleep parameters to further validate the relationship between CDAI and sleep quality. Second, the cross-sectional nature of the research hindered us from drawing a causal relationship between CDAI and sleep duration. Thus, future studies based on longitudinal or prospective cohort designs are warranted to further validate these findings. Third, despite adjusting for a comprehensive set of covariates, including demographic factors, lifestyle habits (e.g., smoking, alcohol use), and health indicators (e.g., BMI, comorbidity status), we cannot rule out the possibility of residual confounding. Unmeasured or imperfectly measured factors, such as chronic stress, detailed aspects of physical activity, undiagnosed sleep apnea, diet quality, socioeconomic status, smoking rates, or other unmeasured health behaviors, may influence both antioxidant intake and sleep quality and could partly account for the observed associations. Chronic social stress—defined as a sustained psychological burden arising from interpersonal relationships, work, finances, or life events—has been consistently linked to sleep quality. Prolonged stress can disrupt sleep architecture and reduce efficiency through activation of the HPA axis, sympathetic nervous system, inflammatory pathways, and emotional-cognitive circuits. Conversely, poor sleep can heighten stress perception, creating a self-reinforcing vicious cycle 79,80. Although the precise expression of this bidirectional relationship may differ across populations because of variations in health status, age, and lifestyle, the overall association remains robust. Unfortunately, a key limitation of the present analysis is that the NHANES database does not contain measures of chronic social stress, preventing us from adjusting for this important confounder. Consequently, the observed link between CDAI and sleep-related outcomes should be validated in longitudinal cohort studies or randomized controlled trials that incorporate chronic social stress (as well as other potential confounders) into the statistical models. Fourth, though we propose a plausible mechanism—CDAI influencing sleep via oxidative stress, inflammation, and the Nrf2 pathway, supported by existing literature—our study lacks relevant biomarker data (e.g., oxidative stress markers MDA/SOD, inflammatory cytokine IL-6) from NHANES. We thus cannot directly validate these mediators in our analysis, with mechanistic interpretations relying only on prior research rather than our cohort's data. Future studies incorporating these biomarkers can directly test the proposed pathway and strengthen causal inference. Fifth, while our study has revealed a statistical correlation between CDAI and sleep, the precise mechanisms by which antioxidant components impact sleep quality remain unclear. Future investigations using multi-omics techniques and specific animal models may help elucidate the specific pathways through which oxidative/antioxidant processes affect sleep quality. Sixth, this study does not adequately address the potential clustering of diet with overall health behaviors, which leads to an interpretation of the relationship between the CDAI and sleep quality that leans toward causality. In reality, higher CDAI scores often co-occur with healthier lifestyles—such as regular sleep schedules, moderate physical activity, lower smoking and drinking frequencies, and better mental well-being—each of which can independently improve sleep. Consequently, CDAI may serve more as a proxy for overall healthy behavior rather than as a direct, independent factor influencing sleep. Without controlling for or stratifying these concurrent health behaviors, the observed CDAI-sleep association could be partially or entirely driven by unmeasured lifestyle differences. Therefore, future research should incorporate more comprehensive lifestyle variables (e.g., physical activity, sleep regularity, stress levels) or employ methods such as propensity-score matching and stratified regression to disentangle CDAI's independent effect from its role as a surrogate for a healthy lifestyle, thereby providing a more nuanced and cautious interpretation. Finally, the present findings are derived exclusively from the NHANES cohort, which predominantly comprises U.S. adults of Western dietary patterns and genetic backgrounds. Consequently, the external generalizability of our results to other populations—particularly non-Western groups with distinct eating habits and genetic profiles—remains uncertain. Future research in diverse international cohorts representing a broader range of regions, ethnicities, cultures, and dietary exposures are needed to validate and extend our results.

## Conclusion

Our study identified a non-linear positive correlation between CDAI and sleep duration among US adults, with a more pronounced association observed in individuals with depression and those taking sleep-affecting medications. Additionally, an inflection point was identified at a CDAI score of 3.2. Beyond this threshold, further increases in CDAI were not associated with additional gains in sleep duration, indicating a saturation effect in which the predicted sleep duration plateaued at approximately 7 hours. To advance these findings and clarify causal mechanisms, future research should prioritize longitudinal cohort studies, mechanistic investigations leveraging multi-omics approaches (e.g., metabolomics, cytokine array techniques, proteomics), and randomized controlled trials integrating dietary antioxidant interventions with objective sleep monitoring (e.g., actigraphy, polysomnography).

## Supplementary Material

Supplementary figure and tables.

## Figures and Tables

**Table 1:** Baseline characteristics of the study population based on CDAI's tertile

Characteristics	Overall	CDAI tertile			P-value
		Low (<-1.60)	Middle (-1.60≤CDAI<1.61)	High (CDAI≥1.61)	
**AGE (years)**	46.00 (32.00-60.00)	46.00 (32.00-61.00)	46.00 (32.00-61.00)	45.00 (32.00-60.00)	0.245
**PIR**	2.52 (1.20-4.64)	1.95 (1.03-3.77)	2.69 (1.27-4.80)	3.06 (1.38-5.00)	<0.001
**BMI (kg/m^2^)**	27.68 (24.15-32.07)	28.00 (24.20-32.50)	27.80 (24.28-32.25)	27.23 (24.00-31.60)	<0.001
**Caffeine (mg)**	109.50 (37.00-215.50)	98.00 (34.50-198.00)	114.00 (40.00-217.50)	118.00 (37.50-228.00)	<0.001
**Energy (Kcal)**	1999.00 (1553.00-2561.50)	1488.50 (1203.00-1827.50)	2027.50 (1705.00-2394.50)	2645.50 (2151.00-3244.50)	<0.001
**Moisture (gm)**	2636.73 (2006.04-3358.13)	2167.04 (1634.73-2887.51)	2702.11 (2058.14-3336.64)	3106.93 (2369.96-3994.16)	<0.001
**Gender**					<0.001
Male	5484 (52.27%)	1366 (39.06%)	1755 (50.19%)	2363 (67.57%)	
Female	5007 (47.73%)	2131 (60.94%)	1742 (49.81%)	1134 (32.43%)	
**Race**					<0.001
Mexican American	1379 (13.14%)	466 (13.33%)	460 (13.15%)	453 (12.95%)	
Other Hispanic	954 (9.09%)	360 (10.29%)	307 (8.78%)	287 (8.21%)	
Non-Hispanic White	5211 (49.67%)	1617 (46.24%)	1771 (50.64%)	1823 (52.13%)	
Non-Hispanic Black	2066 (19.69%)	801 (22.91%)	643 (18.39%)	622 (17.79%)	
Other Race	881 (8.40%)	253 (7.23%)	316 (9.04%)	312 (8.92%)	
**Education**					<0.001
less than high school	1957 (18.65%)	856 (24.48%)	616 (17.62%)	485 (13.87%)	
high school or equivalent	2275 (21.69%)	884 (25.28%)	720 (20.59%)	671 (19.19%)	
college or above	6259 (59.66%)	1757 (50.24%)	2161 (61.80%)	2341 (66.94%)	
**Marital status**					<0.001
Married	5439 (51.84%)	1613 (46.13%)	1863 (53.27%)	1963 (56.13%)	
Never Married	2137 (20.37%)	751 (21.48%)	679 (19.42%)	707 (20.22%)	
Others	2915 (27.79%)	1133 (32.40%)	955 (27.31%)	827 (23.65%)	
**Smoking**					<0.001
Never	5388 (51.36%)	1672 (47.81%)	1845 (52.76%)	1871 (53.50%)	
Former	2673 (25.48%)	769 (21.99%)	929 (26.57%)	975 (27.88%)	
Now	2430 (23.16%)	1056 (30.20%)	723 (20.67%)	651 (18.62%)	
**Alcohol consumption**					<0.001
Mild drinking	5196 (49.53%)	1592 (45.52%)	1756 (50.21%)	1848 (52.85%)	
Moderate drinking	3451 (32.89%)	1250 (35.74%)	1147 (32.80%)	1054 (30.14%)	
Heavy drinking	1844 (17.58%)	655 (18.73%)	594 (16.99%)	595 (17.01%)	
**PA group**					<0.001
Low physical activity	3553 (33.87%)	1414 (40.43%)	1213 (34.69%)	926 (26.48%)	
High physical activity	6938 (66.13%)	2083 (59.57%)	2284 (65.31%)	2571 (73.52%)	
**Diabetes**					<0.001
No	9022 (86.00%)	2933 (83.87%)	3034 (86.76%)	3055 (87.36%)	
Yes	1469 (14.00%)	564 (16.13%)	463 (13.24%)	442 (12.64%)	
**Hypertension**					<0.001
No	6503 (61.99%)	2080 (59.48%)	2175 (62.20%)	2248 (64.28%)	
Yes	3988 (38.01%)	1417 (40.52%)	1322 (37.80%)	1249 (35.72%)	
**CVD**					<0.001
No	9643 (91.92%)	3167 (90.56%)	3210 (91.79%)	3266 (93.39%)	
Yes	848 (8.08%)	330 (9.44%)	287 (8.21%)	231 (6.61%)	
**Cancer**					0.376
No	9509 (90.64%)	3186 (91.11%)	3171 (90.68%)	3152 (90.13%)	
Yes	982 (9.36%)	311 (8.89%)	326 (9.32%)	345 (9.87%)	
**Depression**					<0.001
No	9614 (91.64%)	3065 (87.65%)	3250 (92.94%)	3299 (94.34%)	
Yes	877 (8.36%)	432 (12.35%)	247 (7.06%)	198 (5.66%)	
**Anxiety**					<0.001
No	5822 (55.50%)	1844 (52.73%)	1978 (56.56%)	2000 (57.19%)	
Yes	2038 (19.43%)	799 (22.85%)	653 (18.67%)	586 (16.76%)	
Missing	2631 (25.08%)	854 (24.42%)	866 (24.76%)	911 (26.05%)	
**Medication use**					<0.001
No	8272 (78.85%)	2699 (77.18%)	2735 (78.21%)	2838 (81.16%)	
Yes	2219 (21.15%)	798 (22.82%)	762 (21.79%)	659 (18.84%)	

***Abbreviations:*** CDAI, composite dietary antioxidant index; PA, physical activity; CVD, cardiovascular disease; BMI, body mass index; PIR, ratio of family income to poverty. ***Note:*** Variables including age, PIR, BMI, caffeine, energy, moisture, and CDAI are presented as median (interquartile range, IQR) because of their skewed distribution***.***

**Table 2:** Threshold effect and saturation effect analyses for the association between CDAI and sleep duration

Outcome	β (95%CI) *P*-value
***Model I***	
A straight-line effect	0.02 (0.01, 0.02) 0.0011
***Model II***	
Fold points (K)	3.2
< K-segment effect 1	0.04 (0.02, 0.05) <0.0001
> K-segment effect 2	-0.00 (-0.01, 0.01) 0.8807
Effect size difference of 2 vs. 1	-0.04 (-0.06, -0.02) 0.0002
Equation predicted values at break points	6.95 (6.90, 7.00)
Log likelihood ratio tests	<0.001

***Abbreviations:*** OR, odds ratio; CI, confidence intervals; CDAI, composite dietary antioxidant index. Adjusted for variables of Age, Gender, Race, Education, Marital status, PIR, BMI, Smoking, Drinking, PA, Diabetes, Hypertension, CVD, Cancer, Depression, Anxiety, Pregnancy, Caffeine, Medication Use, Energy, Moisture.

**Table 3:**
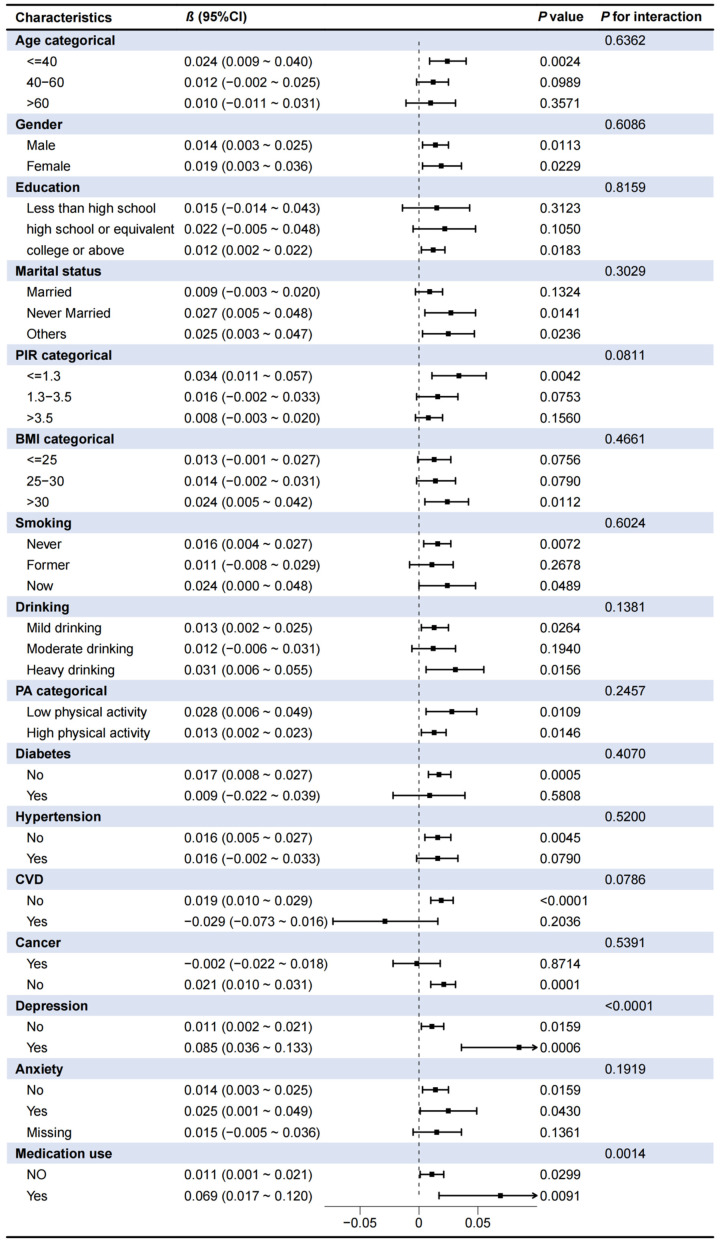
Association between CDAI and sleep duration in different subgroups

**Abbreviations:** CI, confidence intervals; CDAI, composite dietary antioxidant index; PA, physical activity; CVD, cardiovascular disease; BMI, body mass index; PIR, ratio of family income to poverty. The variables adjusted for subgroup analyses were consistent with Model 3 in Table 2 except the stratifying variable.

## Abbreviations

CDAI: composite dietary antioxidant index
BMI: body mass index
PIR: poverty income ratio
CAPI: computer-assisted personal interviewing
