# Supplementary material for: The Sleep Connection: Exploring the Role of Composite Dietary Antioxidant Index in Sleep Quality Based on NHANES 2007-2014
Source: Int J Med Sci. 2026 Jan 1;23(1):227–42. doi: 10.7150/ijms.114874 (PMC12702126; doi:10.7150/ijms.114874)
Supplement: Supplementary file 1 — Supplementary figure and tables. [file ijmsv23p0227s1.pdf]

STable 1. Association between CDAI and sleep quality (related to Figure 2)

Outcomes	Model 1	Model 2	Model 3
Sleep duration, β (95%CI) P-value			
CDAI (continuous)	0.01 (0.01, 0.02) 0.0002	0.01 (0.00, 0.02) 0.0020	0.02 (0.01, 0.02) 0.0011
CDAI (categorical)			
Low	Reference	Reference	Reference
Middle	0.15 (0.08, 0.21) <0.0001	0.12 (0.06, 0.18) 0.0002	0.12 (0.05, 0.19) 0.0005
High	0.16 (0.10, 0.23) <0.0001	0.14 (0.07, 0.20) <0.0001	0.16 (0.08, 0.24) 0.0001
P for trend	<0.0001	<0.0001	<0.0001
Sleep trouble, OR (95%CI) P-value			
CDAI (continuous)	0.97 (0.96, 0.98) <0.0001	0.99 (0.98, 1.00) 0.1712	1.00 (0.98, 1.02) 0.8668
CDAI (categorical)			
Low	Reference	Reference	Reference
Middle	0.86 (0.78, 0.96) 0.0070	0.92 (0.82, 1.03) 0.1422	1.00 (0.88, 1.13) 0.9867
High	0.78 (0.70, 0.87) <0.0001	0.92 (0.82, 1.03) 0.1483	1.03 (0.88, 1.20) 0.7168
P for trend	<0.0001	0.1416	0.7223
Sleep disorder, OR (95%CI) P-value			
CDAI (continuous)	0.99 (0.97, 1.01) 0.3736	0.99 (0.97, 1.01) 0.4105	1.00 (0.97, 1.03) 0.8238
CDAI (categorical)			
Low	Reference	Reference	Reference
Middle	0.98 (0.83, 1.16) 0.8282	0.99 (0.83, 1.18) 0.9190	1.04 (0.86, 1.27) 0.6753
High	0.92 (0.78, 1.10) 0.3561	0.93 (0.78, 1.12) 0.4333	1.01 (0.79, 1.28) 0.9655
P for trend	0.357	0.435	0.954

Abbreviations: OR, odds ratio; CI, confidence intervals; CDAI, composite dietary antioxidant index; PA, physical activity; CVD, cardiovascular disease; BMI, body mass index; PIR, ratio of family income to poverty. ***Note***: Model 1: adjusted for no covariates. Model 2: adjusted for variables of Age, Gender, Race, Education, Marital status, PIR. Model 3: adjusted for variables of Age, Gender, Race, Education, Marital status, PIR, BMI, Smoking, Drinking, PA, Diabetes, Hypertension, CVD, Cancer, Depression, Anxiety, Pregnancy, Caffeine, Medication Use, Energy, Moisture.

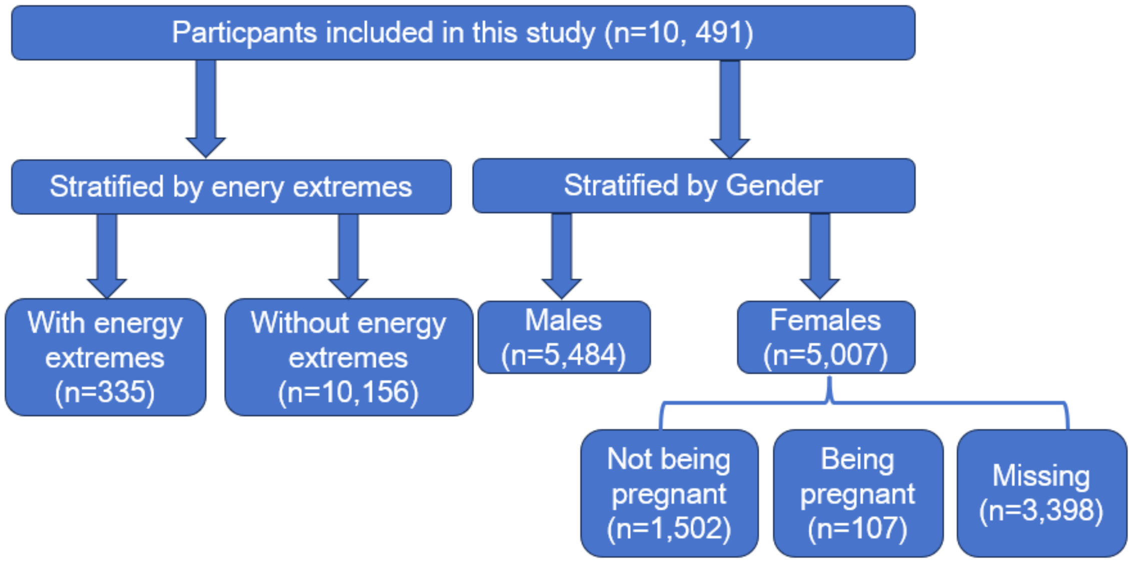

SFigure 1. Participants' characteristics based on variables of gender and energy extremes.

STable 2. Association between CDAI an sleep duration after excluding participants with energy extremes (related to Figure 5A)

Exposure	Model 1<br>*β (95%CI) P-value*	Model 2<br>*β (95%CI) P-value*	Model 3<br>*β (95%CI) P-value*
CDAI	0.013 (0.006, 0.020) 0.00016	0.015 (0.008, 0.022) 0.00003	0.013 (0.006, 0.020) 0.00017
CDAI tertile			
Low	0	0	0
Middle	0.149 (0.085, 0.213) <0.00001	0.156 (0.091, 0.220) <0.00001	0.148 (0.084, 0.212) <0.00001
High	0.162 (0.098, 0.226) <0.00001	0.180 (0.114, 0.246) <0.00001	0.161 (0.097, 0.224) <0.00001
P for trend	<0.00001	<0.00001	<0.00001

Abbreviations: OR, odds ratio; CI, confidence intervals; CDAI, composite dietary antioxidant index; PA, physical activity; CVD, cardiovascular disease; BMI, body mass index; PIR, ratio of family income to poverty. ***Note***: Model 1: adjusted for no covariates. Model 2: adjusted for variables of Age, Gender, Race, Education, Marital status, PIR. Model 3: adjusted for variables of Age, Gender, Race, Education, Marital status, PIR, BMI, Smoking, Drinking, PA, Diabetes, Hypertension, CVD, Cancer, Depression, Anxiety, Pregnancy, Caffeine, Medication Use, Energy, Moisture.

STable 3. Association between CDAI an sleep duration after excluding participants with pregnancy (related to Figure 5B)

Exposure	Model 1<br>*β (95%CI) P-value*	Model 2<br>*β (95%CI) P-value*	Model 3<br>*β (95%CI) P-value*
CDAI	0.032 (0.008, 0.056) 0.00889	0.032 (0.008, 0.055) 0.00920	0.032 (0.008, 0.056) 0.00828
CDAI tertile			
Low	0	0	0
Middle	0.260 (0.103, 0.418) 0.00124	0.260 (0.102, 0.418) 0.00129	0.259 (0.101, 0.417) 0.00135
High	0.196 (0.012, 0.379) 0.03735	0.195 (0.011, 0.379) 0.03812	0.198 (0.014, 0.382) 0.03499
P for trend	0.00915	0.0095	0.00856

Abbreviations: OR, odds ratio; CI, confidence intervals; CDAI, composite dietary antioxidant index; PA, physical activity; CVD, cardiovascular disease; BMI, body mass index; PIR, ratio of family income to poverty. ***Note***: Model 1: adjusted for no covariates. Model 2: adjusted for variables of Age, Gender, Race, Education, Marital status, PIR. Model 3: adjusted for variables of Age, Gender, Race, Education, Marital status, PIR, BMI, Smoking, Drinking, PA, Diabetes, Hypertension, CVD, Cancer, Depression, Anxiety, Pregnancy, Caffeine, Medication Use, Energy, Moisture.
